# Supplementary figures and images for: Efferent projections of topographically distinct relaxin family peptide receptor-3 (RXFP3) lateral hypothalamus/zona incerta cells
Source: Brain Struct Funct. 2026 Jul 15;231(7):97. doi: 10.1007/s00429-026-03146-0 (PMC13372858; doi:10.1007/s00429-026-03146-0)

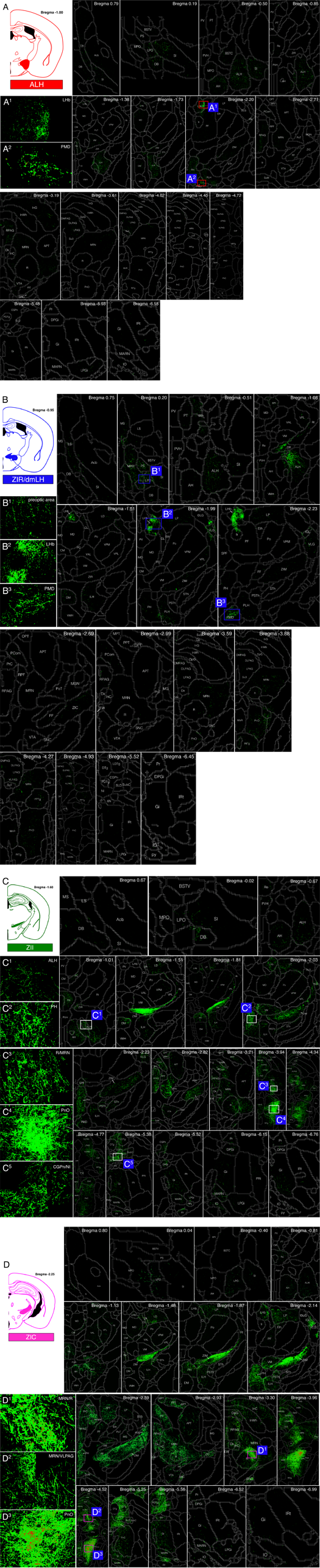

Supplement: Supplementary file 2 — Fig. 4 [file 429_2026_3146_MOESM2_ESM.png]
